# Integrated mRNA and miRNA transcriptome analysis of grape in responses to salt stress

**DOI:** 10.3389/fpls.2023.1173857

**Published:** 2023-05-08

**Authors:** Lingzhu Wei, Yuanpeng Du, Jiang Xiang, Ting Zheng, Jianhui Cheng, Jiang Wu

**Affiliations:** ^1^ Institute of Horticulture, Zhejiang Academy of Agricultural Sciences, Hangzhou, Zhejiang, China; ^2^ College of Horticulture Science and Engineering, Shandong Agricultural University, Taian, Shandong, China

**Keywords:** grapes, transcriptome, salt stress, miRNAs, mRNA

## Abstract

Salt stress is an important factor which may negatively affect plant growth and development. High concentrations of Na^+^ ions can destroy the ion balance in plant somatic cells, as well as destroying cell membranes and forming a large number of reactive oxygen species (ROS) and other damage mechanisms. However, plants have evolved numerous defense mechanisms in response to the damages caused by salt stress conditions. Grape (*Vitis vinifera* L.), a type of economic crop, is widely planted throughout the world. It has been found that salt stress is an important factor affecting the quality and growth of grape crops. In this study, a high-throughput sequencing method was used to identify the differentially expressed miRNAs and mRNAs in grapes as responses to salt stress. A total of 7,856 differentially expressed genes under the salt stress conditions were successfully identified, of which 3,504 genes were observed to have up-regulated expressions and 4,352 genes had down-regulated expressions. In addition, this study also identified 3,027 miRNAs from the sequencing data using bowtie and mireap software. Among those, 174 were found to be highly conserved, and the remaining miRNAs were less conserved. In order to analyze the expression levels of those miRNAs under salt stress conditions, a TPM algorithm and DESeq software were utilized to screen the differentially expressed miRNAs among different treatments. Subsequently, a total of thirty-nine differentially expressed miRNAs were identified, of which fourteen were observed to be up-regulated miRNAs and twenty-five were down-regulated under the salt stress conditions. A regulatory network was built in order to examine the responses of grape plants to salt stress, with the goal of laying a solid foundation for revealing the molecular mechanism of grape in responses to salt stress.

## Introduction

At the present time, salinization is an important factor threatening human survival and development. According to the survey results of Food and Agriculture Organization of the United Nations (FAO), the annual losses caused by land salinization were approximately 110 billion US dollars worldwide (Ismail et al., 2012). In addition, soil salinization also affects the water absorption abilities of crops and the trace elements necessary for growth and development. The long term exposure of plants to salt stress will lead to a series of damages, such as the destruction of cell membrane; metabolic poisoning; formation of a large number of reactive oxygen species; reduced photosynthesis; and reduced nutrient absorption (Hasegawa et al., 2000; Zhu, 2002). In response to the aforementioned adverse stress conditions, plants have built a series of defense mechanisms. These include maintaining water absorbing capacity levels; enhancing the efflux and transportation of salt ions in order to reduce the accumulation of toxins; and transferring sodion into vacuoles to avoid the accumulation of high concentrations of sodion in cytoplasm (Parida and Das, 2005).

In recent years, the molecular mechanisms of plant responses to salt stress have been revealed. A large number of salt stress-related transcription factors, miRNAs, and salt sensitive genes have been consecutively identified. Among those discoveries, salt sensitive genes are a type of gene which plants have utilized to respond to external salt stress. Those identified genes have been observed to show different characteristics in different plant tissue (Kumar et al., 2018; Wani et al., 2020; Wang et al., 2019; Zhao et al., 2021). For example, the salt hypersensitive gene 3 (SOS3) and the sucrose non-fermenting1 (SNF1)-related protein kinase 3 (SnRK3) in Arabidopsis roots were found to be able to respond to the initial osmotic stress signals induced by salt stress (Zhu, 2002). In addition, enhanced responses to ABA 1 (ERA1), protein phosphatase 2C (PP2C), and ABA activated protein kinase (AAPK) and PKS3 can effectively reduce accumulations of toxic ions in plant by delaying stomatal closures during osmotic stress in plants (Zhang et al., 2014). Munns and Tester also found that Na^+^/H^+^ antiporter NHX and VACUOLAR H^+^/^-^ PYROPHOSPHATASE AVP could potentially enhance the absorption capacity of Na^+^ to the vacuoles of root tissue, thereby improving the regulation abilities of plants to cope with osmotic stress (Munns and Tester, 2008). In addition to salt sensitive genes which can directly respond to salt stress, transcription factors which regulate the expressions of those genes also play important roles in plant responses to salt stress conditions (Munns and Tester, 2008). The results revealed that the overexpression of the aforementioned could significantly enhance the tolerance of plants to salt stress (Wani et al., 2020). In addition, the overexpression of the MYB transcription factor (MYB48-1) in rice was observed to enhance the drought and salt stress responses induced by mannitol and propylene glycol (Xiong et al., 2014).

miRNAs are a class of endogenous non-coding small RNAs with a length of approximately 21 bp, which widely exist in both animals and plants (Li et al., 2018). miRNAs can regulate the expressions of downstream genes at the post transcriptional level (Mabuchi et al., 2018; Wang et al., 2020). Increasing amounts of evidences have shown that miRNAs are played important roles in plant responses to salt stress (Yang et al., 2020; Islam et al., 2022). For example, the overexpression of Os-miR528 in creeping bent grass resulted in shortened internodes and increased tillers in transgenic plants. However, the transgenic plants improved salt tolerance by maintaining sodium/potassium balance and improving water holding capacity levels (Yuan et al., 2015). Yang et al. found that Os-miR172c regulates the tolerance of rice to salt stress by participating in stomatal development regulation, stress related gene expressions, and ABA dependent signal transduction processes (Yang et al., 2017). Therefore, the above-mentioned results indicated that miRNAs have major potential for improving crop resistance to salt and alkalization.

Grapes (*Vitis vinifera* L.) are a type of perennial woody vined plant which is widely cultivated throughout the world. The fruit of grape plants are mainly used for wine production, fresh consumption, and raisin processing. Grapes are considered to be an important economic crop. However, grapes are vulnerable to various external environmental factors during cultivation processes. Drought and soil salinization are the main factors affecting grape growth, which may lead to the decreases in fruit quality and limited life spans of the grape plants (Walker et al., 2000; Serra et al., 2014). Since the publication of the grape genome data in 2007, transcriptome analysis has been widely used for the identification of the grape genes involved in salt stress responses (Jaillon et al., 2007; Amirbakhtiar et al., 2021). For example, Sucheta et al. identified 2,793 differentially expressed proteins through the proteomic analysis of the leaves of sensitive grape types (Thomson seedless grapes) and salt resistant grape types (110R-grafted Thomson seedless grapes) at different time points (for example, 6 hours, 48 hours, and 7 days) under salt stress treatments. Among those, 246 differentially expressed proteins were observed to have highly expressive abundance at all time points. Therefore, the results indicated that those proteins may play indispensable roles in the responses of grapes to salt stress (Patil et al., 2019). Das et al. analyzed the mechanism of Thomson seedless grape plant responses to salt stress using a transcriptome analysis approach. The results showed that a large number of genes from the metabolic pathways were regulated by salt stress, including those involved in sugar metabolism, signal transduction, energy metabolism, amino acid metabolism, secondary metabolite synthesis, and lipid metabolism (Das and Majumder, 2019). Therefore, the findings suggested that multiple metabolic processes jointly regulate the responses of grapes to salt stress.

In recent years, the molecular mechanisms of plant responses to salt stress in model plants (such as rice and Arabidopsis) have been revealed. However, since grapes are also considered to be an important economic crop, and soil salinization has become an important factor affecting the yields and quality of grape crops. At the present time, studies regarding the molecular mechanism of the salt tolerance of grapes are relatively scarce. Specifically, research reports investigating the roles of miRNAs in grape responses to salt stress are lacking. In order to address that issue, this study adopted a high-throughput sequencing method to analyze the mRNAs and miRNAs in sample grape leaves at different time points (for example, 0 hours and 7 days) under salt stress treatment conditions. This study explored the related mRNA and miRNAs of grapes in response to salt stress through those analysis methods, and constructed a miRNA-mRNA regulatory network. The goal was to lay a solid foundation for further revealing the molecular mechanism of grape (*Vitis vinifera* L.) responses to salt stress.

## Materials and methods

### Plant sample material and NaCL treatments

In this study, the variety “Kangzhen 3”, which was formed by the hybridization of “Hean 580” and “SO4”, was used as the experimental plant material. During the dormancy stage, cuttings from ‘Kangzhen 3’ were rooted in humid sand crates, and then placed in a controlled culture room (25°C; 90% humidity; 16-hour photoperiod). Young plantlets were further grown in pots containing a soil-peat-sand mixture at 3:1:1 and placed in a controlled greenhouse (20/24°C; 16/8-hour photoperiod) in the greenhouse area of the Zhejiang Academy of Agricultural Sciences. Two months later, the cultured seedlings were treated with 150 mM of NaCl. At the same time, leaf samples were collected at 0 days, 1 day, 3 days, 5 days, and 7 days during the salt stress treatments, respectively. The samples were frozen with liquid nitrogen and stored in a -80 °C refrigerator for this study’s subsequent experimental examinations. The experimental process utilized 7-day salt stressed grape leaves for the miRNAs and transcriptome sequencing analyses, and the 0 hour treatment samples were used as the control.

### RNA isolation

In this study, the Trizol Method (Invitrogen, Carlsbad, CA, USA) was used to isolate total RNA from the grape leaves of the control group and salt stressed groups. A TURBO DNA-free kit (Sigma-Aldrich, St. Louis, MO, USA) was used to extract genomic DNA from the total RNA. The quality and concentration levels of extracted total RNA were measured using an ultraviolet spectrophotometer (Implen, Germany), and used for RNA-Seq and real-time quantitative PCR. In addition, all of the treatments implemented in this study contained three biological replicates.

### Creation and sequencing of a cDNA library

In this experiments, a cDNA library was created using an Ultra™ RNA Library Prep Kit, and the experimental procedures were carried out according to the instructions contained in the kit. The created cDNA library was used for RNA-Seq sequencing. The entire sequencing process was completed by Genepioneer Biotechnologies through Illumina HiSeqTM 2500, and 6 Gb of original data were obtained for each sample. In addition, HISAT and Tophat 2.0.13 software were used to remove the splices and low-quality sequences in the original data, and then map the obtained clean data to the grape genome (Trapnell et al., 2009; Haider et al., 2017). This study utilized Cuffdiff 2.2.1 software (http://cufflinks.cbcb.umd.edu/) to calculate the RPKM/FPKM ratio and predict the expression levels of the different transcripts. Then, the DEGseq2 tool was adopted to analyze the differentially expressed genes. The screening criteria of the differentially expressed genes were FDR < 0.05 and │Log2FC│>1 and it was considered that the gene expression levels had reached the differential level. At the same time, Blast2go software was used to establish the GO terms (GO, http://www.geneontology.org) and the KEGG (https://www.genome.jp/kegg/pathway.html) enrichment analysis.

### Determination of the ion concentration levels

The concentration levels of sodium and potassium ions in the sample grape leaves were measured at different time points of the salt stress treatments. During the experiments, 0.5 g of the samples to be tested were weighed and placed in an oven at 70 °C for drying for 48 hours. Then, the completely dried samples were ground into powder and 5 mL of HNO_3_:HClO_4_ (2:1, volume ratio) mixture were added for the digestive treatments. ICP-MS was used to determine the concentrations of Na^+^, K^+^, and Cl^-^ in the samples (Ma et al., 2015). The concentrations of sodium and potassium ions in the sample grape leaves were measured at different time points during the salt stress treatments. The 0.5 g samples to be tested were weighed and placed in an oven at 70 °C for drying duration of 48 hours.

### Real time quantitative RT-qPCR

In this study, real-time quantitative RT-qPCR was used to analyze the expression levels of some miRNAs under salt stress conditions in order to verify the differentially expressed miRNAs identified by transcriptome. The mature sequences of differentially expressed miRNAs were first searched through the miRBase database and quantitative detection primers were designed. Total RNA was extracted from the sample grape leaves after undergoing 7 days of salt stress conditions using a TRIzol method. The 1 μg purified total RNA was reverse transcribed into single stranded cDNA using a First Stand cDNA Synthesis Kit (Toyobo, Osaka, Japan). Subsequently, the relative expression levels of differential miRNAs were analyzed using an AceQ RT-qPCR SYBR Green Master Mix Kit (Vazyme, Nanjing, China). This study conducted differential miRNAs analyses using a 7900 Real-Time PCR System (Applied Biosystems, Foster City, CA, USA) produced by ABI. The reaction procedure was pre-denaturation at 95°C for ten minutes, and pre-denaturation at 95°C for fifteen seconds, with annealing at 60°C for twenty seconds, and extension at 72°C for thirty seconds. The entire reaction consisted of forty cycles. The internal reference gene used in this study’s experiments was UBC (ubiquitin-conjugating enzyme), and each treatment contained three biological and three technical repeats. The relative expression level was calculated using a 2^−△△Ct^ method.

## Results

### Change trends of the sodium ions, potassium ions, and potassium/sodium ratios at different time points of the salt stress treatments

**Figure 1:**
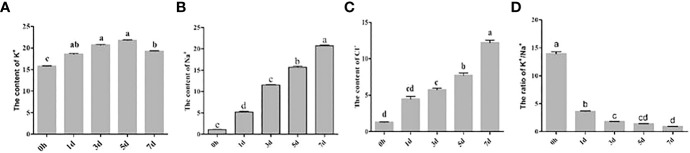
Determination of ion concentrations in grape leaves at different time points of the salt stress treatments. **(A)** K+ **(B)** Na+ **(C)** Cl- **(D)** K+/ Na+.

In this study, the content levels of sodium, potassium, and chloride ions in the sample grape leaves were analyzed at different time points during the salt stress treatments, as shown in **Figure 1**. It was found that the content of sodium and chloride ions in the grape leaves gradually increased with the increase of salt stress treatment time (**Figures 1B, C**), and the concentration levels of potassium ions first increased and then gradually decreased with salt stress treatments (**Figure 1A**). The potassium/sodium ratios reflect the ability of an organism to maintain its ion balance under salt stress. Generally speaking, the higher the potassium/sodium ratio, the stronger the tolerance of an organism to salt stress. In this experiment, the potassium/sodium ratios in the sample grape leaves were observed to gradually decrease over time during the stress treatments (**Figure 1D**). Those findings indicated that the longer the duration of salt stress conditions for the grapes, the more sodium ions accumulate in the plants.

### Expression pattern analysis of the mRNAs

**Figure 2:**
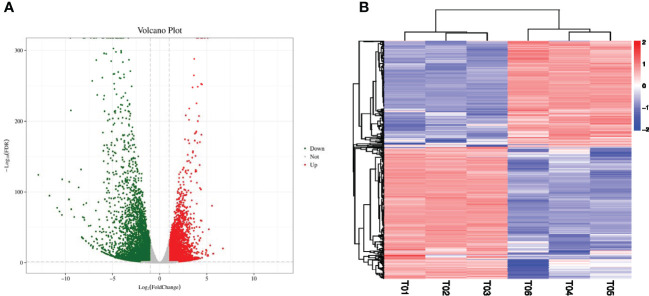
Identification of salt-responsive mRNAs in grapes. **(A)** The volcano plot represents the DE-mRNAs in grapes, and the red and green circles represent the up-regulated and down-regulated mRNAs, respectively. **(B)** The heatmap represents the expression patterns of all mRNAs.

In order to identify the differentially expressed mRNAs in response to the salt stress conditions a high-throughput sequencing method was adopted to sequence and analyze the mRNAs in the sample grape leaves at 0 days and 7 days after salt stress (**Figure 2A**). The numbers of the clean reads obtained by sequencing were as follows: 25,624,885 (K0h-1); 20,904,911 (K0h-2); 23,515,693 (K0h-3); 21,838,361 (K7d-1); 20,904,911 (K7d-2); and 23,515,693 (K7d-3), respectively (**Table 1**). The Q30 values of all the clean reads were more than 90%. Then, through a comparison process, it was ascertained that more than 83% of the sequencing data could be matched to the reference genome. Those matched clean reads were mainly distributed in the exon region, with a coverage of at least 67%. Subsequently, in accordance with the reference genome, splicing of clean reads from the beginning was performed and reconstruction of the transcripts was completed. Finally, 26,346 transcripts were obtained, including approximately 26,252 genes. In order to detect the expression levels of those mRNAs, DESeq software was utilized to calculate the expression levels of the different transcripts, with |log2(Fold Change) |≥1 and FDR < 0.05 as selection criteria. A total of 7,856 differentially expressed genes under the salt stress conditions were successfully identified, of which 3,504 genes were observed to have up-regulated expressions and 4,352 genes had down-regulated expressions (**Figure 2B**).

**Table 1:** Summary of RNA-seq data.

Samples^1^	JSHY-ID	Read number	Base number	GC content	%≥Q30
K0h-1	T01	25624885	7687465500	47.21	93.44
K0h-2	T01	20904911	6271473300	47.15	93.48
K0h-3	T01	23515693	7054707900	47.13	93.72
K7d-1	T01	21838361	6551508300	46.95	93.99
K7d-2	T01	24929391	7478817300	46.69	93.63
K7d-3	T01	26083145	7824943500	47.15	94.15

^1^K0h-1/2/3 and K7d-1/2/3 represent the three replicates of the control groups and the salt-treated groups for grapes, respectively.

### Functional analysis of the DEGs

**Figure 3:**
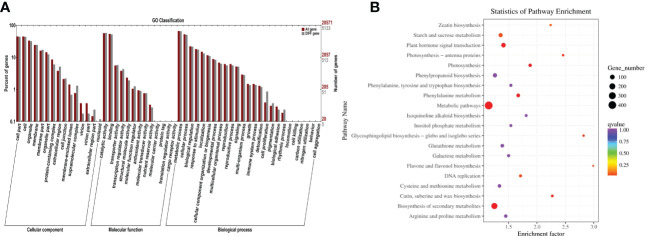
GO and KEGG pathway enrichment analysis results of salt-responsive mRNA target genes. **(A)** GO enrichment results for target genes of DE-mRNAs in grapes. **(B)** KEGG enrichment results for target genes of DE-mRNAs in grapes.

In order to further analyze the function of the aforementioned differentially expressed genes, this study annotated the differentially expressed genes through the non-redundant (NR), GO, Swiss-Prot, KEGG, Pfam, COG, and KOG databases (**Table 2**). As a result, 7,190 annotated genes were identified (**Figure 3A**). According to the GO enrichment analysis, it was found that the majority of the differentially expressed genes were mainly concentrated in the following: Microtubule motor activity (GO:0003777); microtubule binding (GO:0008017); calcium-dependent phospholipid binding (GO:0005544); transferase activity including transferring Hexos (GO:0016758); xyloglucan: xyloglucosyl transferase activity (GO:0016762); S-linalool synthase activity (GO:0034007); quinone binding (GO:0048038), endonuclease activity (GO:0004519); structural constituent of cell wall (GO:0005199); trihydroxystilbene synthase activity (GO:0050350); and other GO terms. The numbers of differentially expressed genes enriched in those GO terms were 86, 119, 27, 562, 40, 13, 38, 257, 58, and 20, respectively. Then, in order to further analyze the possible pathways of the identified differentially expressed genes in metabolism, all the differentially expressed genes were mapped into the KEGG database for KEGG metabolic pathway enrichment. The enrichment results revealed that these differentially expressed genes could be annotated to the metabolic pathways; biosynthesis of secondary metabolites; photosynthesis; phenylalanine metabolism; plant hormone signal transduction; cutin, suberine, and wax biosynthesis; glycosphingolipid biosynthesis-globo and -isoglobo series; photosynthesis-antenna proteins; and flavone and flavonol biosynthesis (**Figure 3B**).

**Table 2:** mRNAs obtained in salt-treated and untreated sRNA libraries.

DEG set	Total	Swiss-Prot	GO	KEGG	COG	KOG	Pfam	NR
K0h_vs_K7d.Mix	7190	5664	5133	1474	2570	3636	5482	7188

### Analysis of the expression patterns of the miRNAs

**Figure 4:**
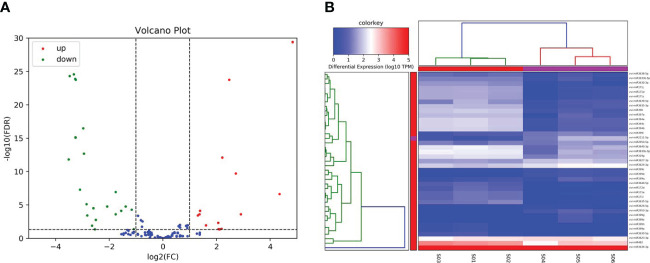
Identification of salt-responsive miRNAs in grapes. **(A)** The volcano plot represents the DE-miRNAs in grapes, and the red and green circles represent the up-regulated and down-regulated mRNAs, respectively. **(B)** The heatmap represents the expression patterns of all mRNAs.

The miRNAs are endogenous non-coding small RNAs which are known to widely exist in both animals and plants. A large number of studies have found that miRNAs play important roles in the growth and development of animals and plants, as well as in the response processes to biological stress. In the present investigation, for the purpose of identifying the miRNAs responding to salt stress in grape plants, a high-throughput sequencing method was adopted to sequence the miRNAs responses to salt stress conditions (**Figure 4A**). The numbers of clean reads obtained by sequencing were as follows: 16,501,599; 17,743,792; 14,646,861; 15,227,202; 14,996,175; and 20,222,375, respectively (**Table 3**), and the Q30 value of the clean reads were greater than 94%. Then, by running a comparison with the reference genome, it was confirmed that at least 77.79% of the clean reads could be matched with the grape genome. At the same time, this study identified 3,027 miRNAs from the sequencing data using bowtie and mireap software. Among those, 174 were found to be highly conserved, and the remainders were less conserved (**Table 4**). In addition, in order to analyze the expression levels of those miRNAs under salt stress conditions, a TPM algorithm and DESeq software were utilized to screen the differentially expressed miRNAs among different treatments. The screening criteria were |log2(FC)|≥1 and Pvalue<0.05. Subsequently, a total of thirty-nine differentially expressed MiRNAs were identified, of which fourteen were observed to be up-regulated miRNAs and twenty-five were down-regulated under the salt stress conditions (**Figure 4B**).

**Table 3:** Statistical analysis of small RNA sequencing data for six samples.

Samples ^1^	JSHY-ID	Read number	Base number	GC content	%≥Q30
K0h-1	S01	16501599	825079950	52.25	96.09
K0h-2	S01	17743792	887189600	52.47	95.39
K0h-3	S01	14646871	732343550	52.36	95.52
K7d-1	S01	15227202	761360100	52.77	94.87
K7d-2	S01	14996175	749808750	53.34	97.14
K7d-3	S01	20222375	1011118750	53.59	97.41

^1^K0h-1/2/3 and K7d-1/2/3 represent the three replicates of the control groups and the salt-treated groups for grapes, respectively.

**Table 4:** Small RNAs obtained in salt-treated and untreated sRNA libraries.

DEG set	Total	Swiss-Prot	GO	KEGG	COG	KOG	Pfam	NR
K0h_vs_K7d.Mix	3155	2699	2542	825	1545	2035	2795	3155

**Figure 5:**
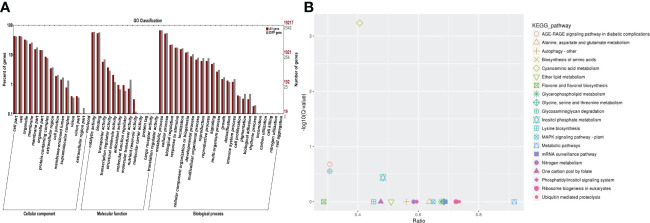
GO and KEGG pathway enrichment analysis results of salt-responsive miRNA target genes. **(A)** GO enrichment results for target genes of DE-miRNAs in grapes. **(B)** KEGG enrichment results for target genes of DE-miRNAs in grapes.

It is well known that miRNAs are involved in many biological processes in which they mainly regulate the expression levels of their downstream target genes. Therefore, psRNATarget was used in this research study to predict the potential target genes of the above-mentioned differential miRNAs. A total of 7,727 potential target genes were successfully predicted. The data of NR, Swiss-Prot, GO, COG, KEGG, KOG, and Pfam were used to annotate the functions of those potential target genes (**Figure 5A**). As a result, 3,155 potential target genes could be annotated. GO and KEGG enrichment analyses of the annotated potential target genes were subsequently performed. It was found that the identified genes were mainly enriched in the following: ATP binding (GO: 0005524); protein serine/threonine kinase activity (GO: 0004674); S-linalool synthase activity (GO: 0034007); calcium-dependent phospholipid binding (GO: 0005544); ADP binding (GO: 0043531); and hydroquinone: oxygen oxidoreductase activity (GO: 0052716), trihydroxystilbene synthase activity (GO: 0050350); microtubule motor activity (GO: 0003777); alliin lyase activity (GO: 0047654); calcium ion binding (GO: 0005509), and other GO terms. The main metabolic pathways of KEGG involved in those potential target genes were as follows: Cyanoamino acid metabolism; inositol phosphate metabolism; glycosaminoglycan degradation; AGE-RAGE signaling pathway in diabetic complications; nitrogen metabolism; MAPK signaling pathway-plant; biosynthesis of amino acids; one carbon pool by folate and mRNA surveillance pathways (**Figure 5B**).

### Validation of the miRNAs in response to salt stress

**Figure 6:**
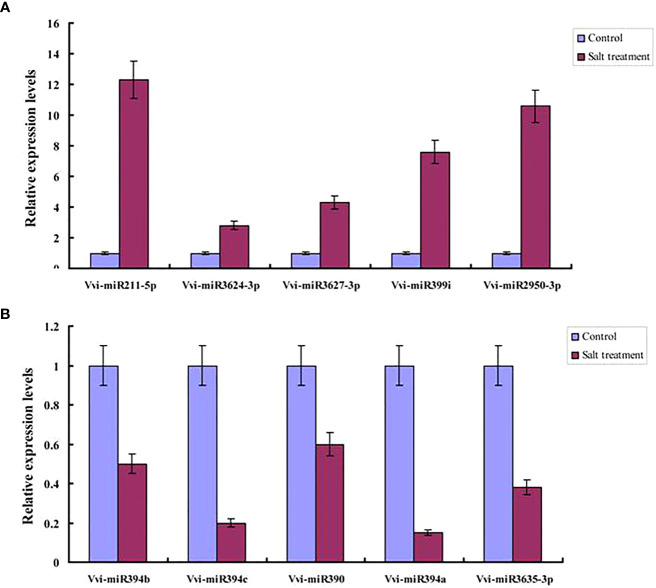
qRT–PCR analysis of upregulated miRNA **(A)** and down-regulated miRNA **(B)** based on RNA-seq.

In order to further verify the differentially expressed miRNAs obtained from the sequencing process, real-time fluorescent qRT-PCR was used to verify the differentially expressed miRNAs. Then, in accordance with the RNA-seq sequencing results, five up-regulated miRNAs (Vvi-miR211-5p, Vvi-miR3624-3p, Vvi-miR3627-3p, Vvi-miR399i, and Vvi-miR2950-3p) and five down-regulated miRNAs (Vvi-miR394b, Vvi-miR394c, Vvi-miR390, Vvi-miR394a, and Vvi-miR3635-3p) were selected under salt stress conditions for the purpose of validation (**Figures 6A, B**). It was found that the expression levels of Vvi-miR211-5p, Vvi-miR3624-3p, Vvi-miR3627-3p, Vvi-miR399i, and Vvi-miR2950-3p had been significantly induced by the salt stress. In addition, it was observed that the expression levels of Vvi-miR211-5p and Vvi-miR2950-3p were much higher than those of the other miRNAs. Furthermore, the expression levels of Vvi-miR394b, Vvi-miR394c, Vvi-miR390, Vvi-miR394a, and Vvi-miR3635-3p were determined to be inhibited by the salt stress conditions, which indicated that salt stress could potentially inhibit the expressions of those miRNAs. Those results were found to be consistent with the expression levels of miRNAs obtained using RNA-seq sequencing. Therefore, it was indicated that the aforementioned miRNAs were played important roles in those processes under salt stress.

## Discussion

With the intensification of human activities and changes to the environment, global soil salinization is gradually increasing. Global soil salinization is an important factor affecting the quality and yields of grapes. It has been found that salt stress can cause many physiological disorders in plants. However, plants can reduce ion toxicity by reducing the concentration levels of Na^+^/H^+^, K^+^, and Cl^-^ (Adams et al., 1993; Brini and Masmoudi, 2012; Kumar et al., 2021). Plant roots absorb Na^+^ and other ions from the soil and transport them to leaves and other organs through transpiration. Under salt stress condition, K^+^ ion channels on the plasma membranes of plant roots reduce the transport of K^+^ ions, but enhance the transport of Na^+^ ions, resulting in imbalances in the ion dynamics of plant cells, thereby leading to cell poisoning. In this study, it was found that at different time points in the salt stress treatments, the K^+^ ion content levels in the sample grape leaf cells displayed a trend of first increasing and then decreasing. Meanwhile, the Na^+^ ion concentrations gradually were observed to increase with the passage of the treatment time (Rajendran et al., 2009). This may have been due to the plants regulating the transport of Na^+^ ions by regulating the expression levels of ions in the channel transport related proteins on cell membrane. The AtNa^+^/H^+^ exchanger-salt sensitive gene SOS1 on the tonoplast of arabidopsis has the ability to promote the excretion of intracellular Na^+^ ions from root cells. However, when the expression levels of that gene are reduced, significant increases in the accumulation of Na^+^ ions in grape plant roots can occur (Qiu et al., 2002; Shi et al., 2002; Nakayama et al., 2022). In addition, researchers have also discovered that by changing the expression levels of the arabidopsis Na^+^ translocator-AtNHX1, the concentration levels of Na^+^ ions in aboveground tissue can be enhanced (Sottosanto et al., 2007). The key factor of salt tolerance in plants is the regulation of the transport of Cl- from the roots to the leaves, or alternatively, maintaining a low level of Cl^-^ ions in the aboveground parts of the plants. However, in order to cope with the high concentrations of Cl^-^ ions in their cells, plants use Cl^-^/H^+^ transporters to maintain low levels of Cl^-^, especially in their aboveground parts. It has been determined that grape plants respond to salt stress by changing the expression levels of those ion channel proteins, salt sensitive proteins, and ion transporters (Wei et al., 2014).

Many stress response genes in plants become activated at both transcriptional and post transcriptional levels in response to salt stress conditions. It is well known that miRNAs are ubiquitous regulators and play important roles in plant responses to biotic and abiotic stress conditions. In recent years, an increasing number of studies have shown that miRNAs play important roles in plant responses to salt stress in Arabidopsis, poplar, soybean, and eggplant (Song et al., 2013; Sun et al., 2015; He et al., 2018; Li et al., 2020; Wen et al., 2020).

In this study, a large proportion of the unigenes showed differential expression levels between salinity stress and control conditions, suggesting that these genes are involved in the grape salinity response. The potential function of these genes that were differentially expressed in response to salinity stress, were similar to those reported for other plants. Several GO terms, catalytic activities, metabolic processes, cellular processes, binding, and cell and cell parts, were observed in this present study (**Figures 2**, **3**, **4**), and these GO terms are generally enriched in response to salt stress in other plants, for example, in salt-stressed roots in soybean (Belamkar et al., 2014), in the leaf tissues of *Petunia hybrida* (Villarino et al., 2014) and in a recretohalophyte, *Reaumuria trigyna* (Dang et al., 2013). Enrichment in these GO terms may indicate plant plasticity in response to salt stress through switches of biochemical and morphological activities in cells. In addition, 39 differentially expressed miRNAs were identified in grape leaves in responses to salt stress, among which the expression levels of miR390 and miR394 were observed to be significantly decreased. In previous related research findings, miR390 and miR394 were also reported to respond to salt stress in other species besides grapes. For example, in Arabidopsis, AtmiR394 responded to salt stress by regulating the expression levels of a downstream target gene which had the ability to encode F-box protein. Furthermore, studies have shown that the LCR gene can participate in the ABA signal transduction pathways of plants. Therefore, it can be assumed that miR394 affects the ABA signal transduction processes in plants by regulating the expression levels of LCR, thereby causing plants to respond to salt stress conditions (Song et al., 2013). The expression levels of miR390 in Jerusalem artichoke plants treated with different concentrations of NaCl showed major differences. The experimental results revealed that under low salt concentration (100 mM) stress treatments, the expression levels of miR390 were significantly increased, thereby reducing the expressions of its target gene-auxin response factor ARF3/4. As a result, the tolerance of the plants to salt was found to be enhanced (Wen et al., 2020). In white poplar, researchers also found that miR390 could participate in the salt tolerance responses of plants and the early root development of plants by regulating the expressions of its target gene auxin response factor ARF3/4 (He et al., 2018). In conclusion, research findings have suggested that miR390 and miR394 may regulate plant responses to salt stress through their downstream genes involved in auxin and ABA signal transduction pathways.

## Conclusion

Soil salinization has become the main factor affecting global crop growth. In the majority of species, there have been reports regarding plant responses to salt stress conditions. Grape is an important economic crop, and salt stress is a key factor affecting the yields and quality of grape crops. However, at the present time, there are few reports available regarding the salt stress responses of grape plants, especially the mechanism of the plant salt tolerance. In this study, a high-throughput sequencing method was adopted in order to identify the differentially expressed mRNAs and miRNAs in response to salt stress. A total of 7,856 differentially expressed genes and thirty-nine differentially expressed miRNAs were identified. These differentially expressed miRNAs and mRNAs were determined to constitute the regulatory network of grape plant responses to salt stress conditions, and participate in the processes of the plant tolerance to salt stress through changes in their expression levels.

## Data availability statement

The original contributions presented in the study are publicly available. This data can be found here: https://www.ncbi.nlm.nih.gov/bioproject/PRJNA949788.

## Author contributions

LW conceived and designed the research; YD, JC performed the experiments; JX analyzed the data and wrote the paper; TZ, LW and JW revised the manuscript. All authors contributed to the article and approved the submitted version.

## References

[B1] Adams P. Thomas J. C. Vernon D. M. Bohnert H. J. Jensen R. G. (1993). Distinct cellular and organismic responses to salt stress. Plant Cell Physiol. 33 (8), 1215. doi: 10.1093/oxfordjournals.pcp.a078376

[B2] Amirbakhtiar N. Ismaili A. Ghaffari M. R. Mirdar Mansuri R. Sanjari S. Shobbar Z. S. (2021). Transcriptome analysis of bread wheat leaves in response to salt stress. PloS One 16 (7), e0254189. doi: 10.1371/journal.pone.0254189 34242309 PMC8270127

[B3] Belamkar V. Weeks N. T. Bharti A. K. Farmer A. D. Graham M. A. Cannon S. B. (2014). Comprehensive characterization and RNA-seq profiling of the HD-zip transcription factor family in soybean *(Glycine max*) during dehydration and salt stress. BMC Genomics 15, 950. doi: 10.1186/1471-2164-15-950 25362847 PMC4226900

[B4] Brini F. Masmoudi K. (2012). Ion transporters and abiotic stress tolerance in plants. ISRN Mol. Biol. 2012, 927436. doi: 10.5402/2012/927436 27398240 PMC4907263

[B5] Dang Z. H. Zheng L. L. Wang J. Gao Z. Wu S. B. Qi Z.. (2013). Transcriptomic profiling of the salt-stress response in the wild recretohalophyte *Reaumuria trigyna* . BMC Genomics 14, 29. doi: 10.1186/1471-2164-14-29 23324106 PMC3562145

[B6] Das P. Majumder A. L. (2019). Transcriptome analysis of grapevine under salinity and identification of key genes responsible for salt tolerance. Funct. Integr. Genomics 19 (1), 61–73. doi: 10.1007/s10142-018-0628-6 30046943

[B7] Haider M. S. Kurjogi M. M. Khalil-Ur-Rehman M. Fiaz M. Pervaiz T. Jiu S.T.. (2017). Grapevine immune signaling network in response to drought stress as revealed by transcriptomic analysis. Plant Physiol. Biochem. 121, 187–195. doi: 10.1016/j.plaphy.2017.10.026 29127881

[B8] Hasegawa P. M. Bressan R. A. Zhu J. K. Bohnert H. J. (2000). Plant cellular and molecular responses to high salinity. Annu. Rev. Plant Physiol. Plant Mol. Biol. 51, 463–499. doi: 10.1146/annurev.arplant.51.1.463 15012199

[B9] He F. Xu C. Fu X. Shen Y. Guo L. Leng M.. (2018). The MicroRNA390/TRANS-ACTING SHORT INTERFERING RNA3 module mediates lateral root growth under salt stress *via* the auxin pathway. Plant Physiol. 177 (2), 775–791. doi: 10.1104/pp.17.01559 29717017 PMC6001319

[B10] Islam W. Waheed A. Naveed H. Zeng F. (2022). MicroRNAs mediated plant responses to salt stress. Cells 11 (18), 2806. doi: 10.3390/cells11182806 36139379 PMC9496875

[B11] Ismail A. Riemann M. Nick P. (2012). The jasmonate pathway mediates salt tolerance in grapevines. J. Exp. Bot. 63 (5), 2127–2139. doi: 10.1093/jxb/err426 22223808 PMC3295401

[B12] Jaillon O. Aury J. M. Noel B.. (2007). The grapevine genome sequence suggests ancestral hexaploidization in major angiosperm phyla. Nature. 449 (7161), 463–467. doi: 10.1038/nature06148 17721507

[B13] Kumar V. Khare T. Shriram V. Wani S. H. (2018). Plant small RNAs: the essential epigenetic regulators of gene expression for salt-stress responses and tolerance. Plant Cell Rep. 37 (1), 61–75. doi: 10.1007/s00299-017-2210-4 28951953

[B14] Kumar S. Li G. Yang J. Huang X. Ji Q. Liu Z.. (2021). Effect of salt stress on growth, physiological parameters, and ionic concentration of water dropwort *(Oenanthe javanica*) cultivars. Front. Plant Sci. 12. doi: 10.3389/fpls.2021.660409 PMC8256277 34234795

[B15] Li J. Cui J. Dai C. Liu T. Cheng D. Luo C. (2020). Whole-transcriptome RNA sequencing reveals the global molecular responses and CeRNA regulatory network of mRNAs, lncRNAs, miRNAs and circRNAs in response to salt stress in sugar beet *(Beta vulgaris*). Int. J. Mol. Sci. 22 (1), 289. doi: 10.3390/ijms22010289 33396637 PMC7795855

[B16] Li Z. Q. Xu R. D. Li N. (2018). MicroRNAs from plants to animals, do they define a new messenger for communication? Nutr. Metab. 15, 68. doi: 10.1186/s12986-018-0305-8 PMC6167836 30302122

[B17] Ma Y. Wang J. Yan Z. Geng F. Cramer G. R. Cheng Z. M. (2015). Subfunctionalization of cation/proton antiporter 1 genes in grapevine in response to salt stress in different organs. Horticulture Res. 2 (7), 15031. doi: 10.1038/hortres.2015.31 PMC4591679 26504576

[B18] Mabuchi K. Maki H. Itaya T. Suzuki T. Nomoto M. Sakaoka S. (2018). MYB30 links ROS signaling, root cell elongation, and plant immune responses. Proc. Natl. Acad. Sci. USA 115 (20), E4710–E4719. doi: 10.1073/pnas.1804233115 29712840 PMC5960331

[B19] Munns R. Tester M. (2008). Mechanisms of salinity tolerance. Annu. Rev. Plant Biol. 59, 651–681. doi: 10.1146/annurev.arplant.59.032607.092911 18444910

[B20] Nakayama R. Safi M. T. Ahmadzai W. Sato K. Kawaura K. (2022). Comparative transcriptome analysis of synthetic and common wheat in response to salt stress. Sci. Rep. 12 (1), 11534. doi: 10.1038/s41598-022-15733-2 35798819 PMC9262916

[B21] Parida A. K. Das A. B. (2005). Salt tolerance and salinity effects on plants: a review. Ecotoxicol Environ. Saf. 60 (3), 324–349. doi: 10.1016/j.ecoenv.2004.06.010 15590011

[B22] Patil S. Shinde M. Prashant R. Kadoo N. Upadhay A. Gupta V. (2019). Comparative proteomics unravels the differences in salt stress response of own rooted and grafted thompson seedless grapevines. J. Proteome Res. 19, 583–599. doi: 10.1021/acs.jproteome.9b00420 31808345

[B23] Qiu Q. S. Guo Y. Dietrich M. A. Schumaker K. S. Zhu J. K. (2002). Regulation of SOS1, a plasma membrane Na+/H+ exchanger in arabidopsis thaliana, by SOS2 and SOS3. Proc. Natl. Acad. Sci. U.S.A. 99 (12), 8436–8441. doi: 10.1073/pnas.122224699 12034882 PMC123085

[B24] Rajendran K. Tester M. Roy S. J. (2009). Quantifying the three main components of salinity tolerance in cereals. Plant Cell Environ. 32 (3), 237–249. doi: 10.1111/j.1365-3040.2008.01916.x 19054352

[B25] Serra I. Strever A. Myburgh P. A. Deloire A. (2014). Review: the interaction between rootstocks and cultivars *(Vitis Vinifera* l.) to enhance drought tolerance in grapevine. Aust. J. Grape Wine Res. 20, 1–14. doi: 10.1111/ajgw.12054

[B26] Shi H. Quintero F. J. Pardo J. M. Zhu J. K. (2002). The putative plasma membrane Na(^+^)/H(^+^) antiporter SOS1 controls long-distance na(^+^) transport in plants. Plant Cell. 14 (2), 465–477. doi: 10.1105/tpc.010371 11884687 PMC152925

[B27] Song J. B. Gao S. Sun D. Li H. Shu X. X. Yang Z. M. (2013). miR394 and LCR are involved in arabidopsis salt and drought stress responses in an abscisic acid-dependent manner. BMC Plant Biol. 13, 210. doi: 10.1186/1471-2229-13-210 24330668 PMC3870963

[B28] Sottosanto J. B. Saranga Y. Blumwald E. (2007). Impact of AtNHX1, a vacuolar Na+/H+ antiporter, upon gene expression during short- and long-term salt stress in *Arabidopsis thaliana* . BMC Plant Biol. 7, 18. doi: 10.1186/1471-2229-7-18 17411438 PMC1853094

[B29] Sun X. Xu L. Wang Y. Yu R. Zhu X. Luo X.. (2015). Identification of novel and salt-responsive miRNAs to explore miRNA-mediated regulatory network of salt stress response in radish *(Raphanus sativus* l.). BMC Genomics 16 (1), 197. doi: 10.1186/s12864-015-1416-5 25888374 PMC4381364

[B30] Trapnell C. Pachter L. Salzberg S. L. (2009). TopHat: discovering splice junctions with RNA-seq. Bioinformatics. 25 (9), 1105–1111. doi: 10.1093/bioinformatics/btp120 19289445 PMC2672628

[B31] Villarino G. H. Bombarely A. Giovannoni J. J. Scanlon M. J. Mattson N. S. (2014). Transcriptomic analysis of petunia hybrida in response to salt stress using high throughput RNA sequencing. PloS One 9, e94651. doi: 10.1371/journal.pone.0094651 24722556 PMC3983219

[B32] Walker R. R. Read P. E. Blackmore D. H. (2000). Rootstock and salinity effects on rates of berry maturation, ion accumulation and colour development in Shiraz grapes. Aust. J. Grape Wine Res. 6 (3), 227–239. doi: 10.1111/j.1755-0238.2000.tb00183.x

[B33] Wang J. Ye Y. Xu M. Fen L. Xu L. (2019). Roles of the SPL gene family and miR156 in the salt stress responses of tamarisk *(Tamarix chinensis)* . BMC Plant Biol. 19, 370. 31438851 10.1186/s12870-019-1977-6 PMC6704519

[B34] Wang J. Ye Y. Xu M. Fen L. Xu L. (2020). Comparative analysis of salt responsive gene regulatory networks in rice and arabidopsis. Comput. Biol. Chem. 85, 107188. doi: 10.1016/j.compbiolchem.2019.107188 31954202

[B35] Wang R. Cheng Y. Ke X. Zhang X. Zhang H. Huang J. (2020). Comparative analysis of salt responsive gene regulatory networks in rice and arabidopsis. Comput. Biol. Chem. 85, 107188. doi: 10.1016/j.compbiolchem.2019.107188 31954202

[B36] Wani S. H. Kumar V. Khare T. Guddimalli R. Parveda M. Solymosi K. (2020). Engineering salinity tolerance in plants: progress and prospects. Planta. 251 (4), 76. doi: 10.1007/s00425-020-03366-6 32152761

[B37] Wei L. I. Wang L. Cao J. Yu B. J. (2014). Bioinformatics analysis of CLC homologous genes family in soybean genome. J. Nanjing Agric. Univ. 37, 35–43. doi: 10.7685/j.issn.1000-2030

[B38] Wen F. L. Yue Y. He T. F. Gao X. M. Zhou S. Z. Xiao H. L. (2020). Identification of miR390-TAS3-ARF pathway in response to salt stress in *Helianthus tuberosus* l. Gene 738, 144460. doi: 10.1016/j.gene.2020.144460 32045659

[B39] Xiong H. Li J. Liu P. Gao X. M. Zhou S. Z. Xiao H. L. (2014). Overexpression of OsMYB48-1, a novel MYB-related transcription factor, enhances drought and salinity tolerance in rice. PloS One 9 (3), e92913. doi: 10.1371/journal.pone.0092913 24667379 PMC3965499

[B40] Yang W. Fan T. Hu X. Cheng T. H. Zhang M. Y. (2017). Overexpressing osa-miR171c decreases salt stress tolerance in rice. J. Plant Biol. 60 (5), 485–492. doi: 10.1007/s12374-017-0093-0

[B41] Yang Z. Zhu P. Kang H. Liu L. Cao Q. Sun J.. (2020). High-throughput deep sequencing reveals the important role that microRNAs play in the salt response in sweet potato *(Ipomoea batatas* l.). BMC Genomics 21 (1), 164. doi: 10.1186/s12864-020-6567-3 32066373 PMC7027035

[B42] Yuan S. Li Z. Li D. Yuan N. Hu Q. Luo H. (2015). Constitutive expression of rice MicroRNA528 alters plant development and enhances tolerance to salinity stress and nitrogen starvation in *Creeping Bentgrass* . Plant Physiol. 169 (1), 576–593. doi: 10.1104/pp.15.00899 26224802 PMC4577425

[B43] Zhang T. Chen S. Harmon A. C. (2014). Protein phosphorylation in stomatal movement. Plant Signal Behav. 9 (11), e972845. doi: 10.4161/15592316.2014.972845 25482764 PMC4622631

[B44] Zhao S. Zhang Q. Liu M. Zhou H. Ma C. Wang P. (2021). Regulation of plant responses to salt stress. Int. J. Mol. Sci. 22 (9), 4609. doi: 10.3390/ijms22094609 33924753 PMC8125386

[B45] Zhu J. K. (2002). Salt and drought stress signal transduction in plants. Annu. Rev. Plant Biol. 53, 247–273. doi: 10.1146/annurev.arplant.53.091401.143329 12221975 PMC3128348

